# Targeted metabolomic profiling of RAW 264.7 cells infected with *Brucella canis* identifies time-dependent metabolic changes

**DOI:** 10.1371/journal.pone.0353470

**Published:** 2026-07-16

**Authors:** Woo Bin Park, Su Min Kyung, Suji Kim, Young Ju Lee, Han Sang Yoo

**Affiliations:** 1 Department of Infectious Diseases, College of Veterinary Medicine, Seoul National University, Seoul, Republic of Korea; 2 Research Institute for Veterinary Science and BK21, Seoul National University, Seoul, Republic of Korea; 3 College of Veterinary Medicine, Kyungpook National University, Daegu, Republic of Korea; East Carolina University Brody School of Medicine, UNITED STATES OF AMERICA

## Abstract

Canine brucellosis caused by *Brucella canis* is gaining public-health relevance as companion-dog ownership and close human–animal contact increase. Yet mechanistic insight into *B. canis* host responses remains limited at the pathway level. To address this gap, we profiled infection-associated metabolic responses in RAW 264.7 cells across multiple post-infection time points using targeted metabolomics (CE–MS). We observed a coherent, time-ordered pattern with the largest shifts at 24 h: arginine, hydroxyproline, choline, cysteine, β-alanine, and glycerol-3-phosphate showed pronounced decreases, and tyrosine decreased modestly; α-ketoglutarate displayed a lower mean at 12 h. Unsupervised analyses separated infected and control groups over time (PCA PC1 = 59.4%, PC2 = 16.7%; 76.1% cumulative), providing an orthogonal summary of the temporal divergence. A supplementary sensitivity analysis (Minimum Cell Loss) was included as exploratory contextual information regarding potential cell-number effects, although direct measurements of infection efficiency and macrophage viability were not performed. An integrated pathway schematic summarizes metabolite associations related to nitric-oxide–related, mitochondrial/energy, redox/glutathione, extracellular-matrix, membrane-lipid, and neuroimmune-related pathways. Together, these results deliver a time-resolved view of *B. canis*–macrophage metabolic responses and delineate prioritized avenues for targeted mechanistic validation.

## Introduction

Brucellosis is a zoonotic infectious disease caused by intracellular gram-negative bacteria belonging to the genus *Brucella*, that can establish chronic infections in both humans and various animal species [1]. In animals, brucellosis commonly leads to reproductive disorders such as abortion, infertility, orchitis, and epididymitis, posing significant challenges to livestock and companion animal management [2,3]. However, human brucellosis is characterized by nonspecific symptoms including recurrent fever, fatigue, joint pain, and neurological complications, making diagnosis and treatment particularly challenging and emphasizing the importance of disease in public health [4,5]. Among the various species of *Brucella*, canine brucellosis caused by *Brucella canis* has been increasingly recognized as an important zoonotic pathogen characterized by persistent infections that have serious implications for both veterinary and public health. However, despite its significant threat, canine brucellosis remains understudied compared with infections with other *Brucella* species, such as *B. abortus*, *B. suis*, and particularly *B. melitensis*, the latter being widely recognized for its pathogenicity and public health importance [6–9]. This gap in knowledge hinders the development of effective disease management strategies, emphasizing more detailed investigations into the specific pathogenic mechanisms associated with *B. canis* infections.

The ability of *Brucella spp*. to survive and replicate within host macrophages is central to their pathogenesis, allowing the bacteria to evade early immune responses and establish persistent infections [7,10]. Previous studies have shown that intracellular pathogens, including various *Brucella* species, strategically manipulate host cellular metabolism and signaling pathways to create an intracellular niche favorable for their survival and replication [11,12]. However, systematic investigations into the host metabolic alterations specifically triggered by *B. canis* infection have not been adequately conducted, leaving a substantial gap in our understanding of canine brucellosis pathogenesis.

Metabolomics, which involves comprehensive analysis of cellular metabolites, has become a powerful tool for elucidating the complex interactions between pathogens and their hosts [13–15]. Metabolic reprogramming in host cells is increasingly recognized as a crucial pathogenic mechanism employed by intracellular bacteria to modulate host immune responses, antioxidant defenses, and energy metabolism, thereby facilitating their intracellular survival [11,16,17]. Recent studies have shown that pathogens strategically alter host metabolic pathways not only to evade immune detection but also to create a favorable intracellular environment that supports their replication and long-term persistence [17]. Although numerous studies have employed metabolomics to investigate host‒pathogen interactions involving bacterial infections such as *Mycobacterium tuberculosis*, highlighting critical disruptions in host energy metabolism, antioxidant defense, and immune response pathways [11,18], studies specifically addressing the metabolic or transcriptomic dynamics of macrophages infected by *B. canis* remain scarce. A recent transcriptomic analysis of DH82 canine macrophages infected with *B. canis* revealed significant upregulation of innate immune pathways, including those related to dendritic cell maturation, TLR, and TREM1 signaling, and simultaneous downregulation of lipid metabolism pathways during early infection [19]. Additionally, recent research by Zhang et al. (2024) [20] using RAW264.7 macrophages demonstrated that *Brucella* infection manipulates host cell metabolism through suppression of the GPX4–GSH axis, thereby inducing ferroptosis pathways and facilitating bacterial intracellular replication and egress. Nevertheless, targeted metabolomic profiling specifically addressing the metabolic dynamics of macrophage in response to *B. canis* infection has yet to be conducted, highlighting the novelty, importance, and clear necessity of the present study.

This study aims to address this critical gap by providing the first comprehensive characterization of host metabolic alterations induced by *B. canis* infection in RAW 264.7 cells. Notably, our analysis highlights previously unreported metabolic changes involving glutathione synthesis, mitochondrial energy metabolism, and neuroimmune interactions mediated by alterations in tyrosine and choline metabolism [21,22]. To our knowledge, this study provides one of the first targeted metabolomic descriptions of RAW 264.7 macrophages following *B. canis* infection and may provide a preliminary framework for future comparative studies involving *Brucella* species and other intracellular pathogens.

Ultimately, the metabolite patterns identified through this targeted metabolomics approach may contribute to a broader understanding of host–pathogen metabolic interactions during *B. canis* infection and provide a framework for future mechanistic investigation.

## Materials and methods

### Ethics statement

This study was conducted in an approved facility in strict accordance with all university and federal regulations. All the experiments were reviewed and approved by the Seoul National University Institutional Biosafety Committee (protocol: SNUIBC-R180912-3).

All experiments described in this study were conducted following approval from the Institutional Biosafety Committee (IBC) of Jeonbuk National University (approval number JBNU 2022-04-001) and performed in a Biosafety Level 3 (BSL-3) laboratory at the Institute of Zoonosis, Jeonbuk National University.

### Cell culture and infection with *B. canis*

The murine macrophage cell line RAW 264.7 (ATCC, Manassas, VA, USA) was cultured in Dulbecco’s modified Eagle medium (DMEM; Gibco, Grand Island, NY, USA) supplemented with 10% fetal bovine serum (FBS; Gibco), 100 U/mL penicillin, and 100 µg/mL streptomycin (Gibco) at 37 °C in a humidified atmosphere containing 5% CO₂. For infection assays, the cells were harvested using gentle scraping, centrifuged at 200 × g for 5 min, and resuspended in fresh medium. Viable cell numbers were determined using trypan blue exclusion assay and a hemocytometer. The macrophages were subsequently seeded at an identical density of approximately 1.5 × 10^6^ viable cells per well in 6-well plates and allowed to adhere overnight before infection.

The cell infection experiments were conducted in a biosafety level 3 (BSL-3) laboratory at the Institute of Zoonosis, Jeonbuk National University. Prior to infection, confluence and viability were microscopically confirmed to ensure uniformity across experimental conditions. Although cell number and morphology were visually confirmed, no quantitative measures of infection efficiency such as CFU counts or fluorescence-based infection assays were performed.

*B. canis* strain RM6/66 was cultured in Brucella broth (Becton, Dickinson and Company, Sparks, MD, USA) at 37 °C under 5% CO₂ until it reached the exponential growth phase (approximately 3.06 × 10^9^ CFU/mL). Bacterial cells were harvested by centrifugation, washed twice in sterile phosphate-buffered saline (PBS; Gibco), and adjusted to a multiplicity of infection (MOI) of 100 bacteria per macrophage.

After infection, the cells were incubated for 2 hours at 37 °C with 5% CO₂, and the extracellular bacteria were subsequently eliminated by treatment with gentamicin (50 µg/mL; Sigma‒Aldrich, St. Louis, MO, USA) for 30 minutes, followed by additional washing steps with PBS [6,7].

For metabolomics analysis, both infected and uninfected cells were collected at different time points: 0 hours (immediately after infection and washing), 2 hours, 12 hours, and 24 hours post-infection. Each experimental condition was performed in triplicate.

### Metabolite extraction

Intracellular metabolites were extracted following previously established CE‒MS metabolomics protocols described by Soga et al. [23–25] with minor modifications (Human Metabolome Technologies Protocol, 2022). Briefly, after the culture medium was removed, the macrophages were quickly washed twice with a 5% mannitol solution (Sigma‒Aldrich) at room temperature to eliminate extracellular metabolites. The cells were immediately lysed with 800 µL of LC‒MS‒grade methanol (Wako Chemicals, Osaka, Japan) at room temperature for 30 s. Subsequently, 550 µL of internal standard solution (Human Metabolome Technologies, Yamagata, Japan), diluted 1,000-fold with Milli-Q water, was added, and the mixture was gently homogenized by pipetting. A total volume of 1,000 µL of cell lysate extract was transferred to pre-chilled 1.5-mL microtubes and placed on ice until further experiments were performed (Human Metabolome Technologies Protocol, 2022). The cell lysates were subsequently centrifuged at 2,300 × g for 5 min at 4 °C to remove cellular debris. The clear supernatant was carefully transferred into two tubes of 350 µL each into prewashed centrifugal filter units (Ultrafree-MC PLHCC, HMT, Japan). Ultrafiltration was conducted at 9,100 × g at 4 °C for approximately 5 hours until the filtrates had completely passed through the filter units. Filtrates were then collected for subsequent vacuum evaporation and concentration (Human Metabolome Technologies Protocol, 2022). The filtered samples were completely dried using a centrifugal evaporator (Eyela CVE-3100, Tokyo, Japan) operating under vacuum conditions at 1,500 rpm and 1,000 Pa at room temperature for 2 hours. The dried samples were sealed with parafilm and stored at −80 °C until CE − MS analysis. For transportation and subsequent analysis, samples were packed in dry ice to maintain sample integrity (Human Metabolome Technologies Protocol, 2022).

### Capillary electrophoresis–mass spectrometry (CE − MS) analysis

Metabolomic analysis was performed using capillary electrophoresis time-of-flight mass spectrometry (CE-TOFMS; Agilent Technologies, Santa Clara, CA, USA) according to the manufacturer's instructions with minor modifications. Briefly, metabolite extracts were dissolved in ultrapure water containing reference compounds. CE-MS was conducted using fused silica capillaries (50 µm i.d. × 100 cm total length) filled with electrolyte solutions optimized for cation and anion analyses. Metabolites were separated at a voltage of 27 kV for cations and 30 kV for anions. Accurate mass data acquisition was achieved with TOF − MS operating in positive and negative ionization modes. Data processing, peak extraction, and normalization against internal standards were performed using MasterHands software (Keio University, Japan), with metabolite annotation based on migration time (MT) and mass-to-charge (m/z) ratios matched to the HMT metabolite database (Soga et al., 2000, 2002, 2003).

### Statistical analysis and pathway mapping

Group comparisons were performed per time point (Infected vs Control; *n* = 3 per group) using Welch’s t‑test (two‑sided) on replicate‑level values; multiple testing was controlled by Benjamini–Hochberg FDR (reporting *q*; threshold *q* < 0.05). We report ratio, log2FC, *p* and *q* in Supplementary S3 Table. Quantified values are reported as pmol/10^6^ cells. For multivariate analyses, PCA and hierarchical clustering were computed in MetaboAnalyst 5.0 after row‑wise z‑scoring of metabolites; features present in ≥80% of samples were retained and occasional missing values were imputed by metabolite‑wise means. PCA explained variance and sample scores are provided in S4 Table, and the complete heatmap matrix with sample annotations is provided in S5 Table. Because infection efficiency/viability were not directly measured, we additionally performed an upper‑bound sensitivity analysis (Minimum Cell Loss, MCL = 1 − mean_Infected/mean_Control) to gauge the extent to which cell‑number differences alone could account for observed decreases (S6 Table). Integrated pathway schematics were prepared in VANTED as descriptive visualizations without additional statistical inference.

### Data Organization

All replicate-level quantified values and per-timepoint summary statistics (Welch’s t-tests with Benjamini–Hochberg FDR; reporting p and q) are provided in Supplementary S3 Table. Complete sample metadata (IDs, group/time point labels, QC flags) are summarized in Supplementary S1 Table, and the compound/annotation list used for targeted assignment is provided in Supplementary S2 Table. The Minimum Cell Loss (MCL) sensitivity table computed from replicate-level values is provided in Supplementary S6 Table.

## Results

Targeted CE–MS profiling revealed time-dependent differences in RAW 264.7 cells following *B. canis* infection. The largest effects appeared at 24 h, where several quantified metabolites were lower in infected cells relative to controls. Formal per-timepoint comparisons (Infected vs Control; n = 3 per group) used Welch’s t-tests with Benjamini–Hochberg FDR; full replicate-level values and summary statistics are provided in Table 1 and Supplementary S3 Table. At 24 h, treatment/control ratios (T/C) were approximately arginine 0.25, hydroxyproline 0.24, choline 0.30, cysteine 0.32, β-alanine 0.40, glycerol-3-phosphate 0.47, and tyrosine 0.68; α-ketoglutarate lacked sufficient replicate coverage at 24 h, with a lower mean observed at 12 h (ratio ≈0.67).

**Table 1:** Selected metabolites at 24 h (Infected vs Control). Unit: pmol/10^6^ cells. Statistics: Welch’s *t*-test (two‑sided) with Benjamini–Hochberg FDR (reporting *q*). Full replicate-level values and statistics are provided in Supplementary S3 Table.

Metabolite	Pathway & biological role	Control (mean)	Infected (mean)	Ratio (T/C)	log2FC	*p*	*q*
**Arginine**	Immune modulation; nitric oxide synthesis (antimicrobial activity)	181.28	44.78	0.247	−2.017	0.0697	0.7619
**Tyrosine**	Neurotransmission; precursor for dopamine and norepinephrine synthesis	6.19	4.22	0.682	−0.552	0.2055	0.7619
**β-Alanine**	Energy metabolism; essential for coenzyme A synthesis	156.37	61.85	0.396	−1.338	0.1995	0.7619
**Cysteine**	Antioxidant defense; precursor for glutathione synthesis	40.43	12.92	0.319	−1.646	0.0894	0.7619
**Hydroxyproline**	Extracellular matrix; collagen stabilization and tissue remodeling	267.48	63.36	0.237	−2.078	0.1052	0.7619
**Choline**	Cell signaling; precursor for phosphatidylcholine synthesis	7.72	2.31	0.300	−1.739	0.2136	0.7619
**Glycerol-3-phosphate**	Lipid metabolism; intermediate in glycerolipid synthesis and energy metabolism	40.55	18.99	0.468	−1.094	0.1815	0.7619

α-Ketoglutarate (24 h) was not reported due to insufficient replicate coverage; see the 12 h trend in Supplementary S3 Table. q-values were computed across all quantified metabolites within the same time point.

**Fig 1:**
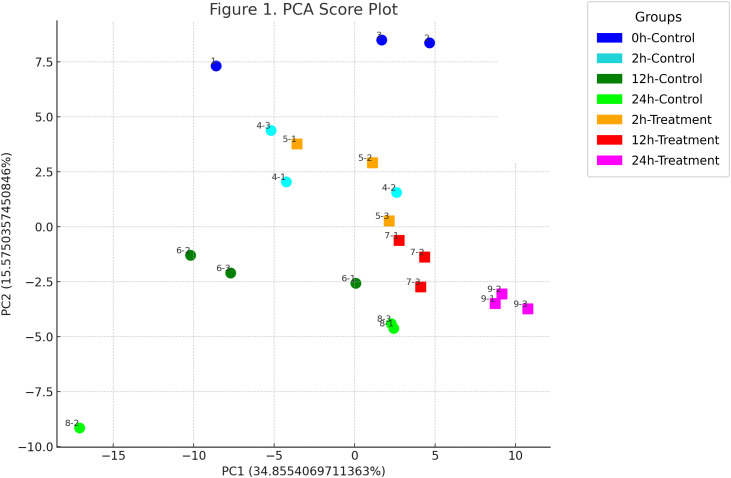
Principal Component Analysis (PCA) Score plot illustrating metabolic profiles. Scores plot shows separation between infected and control RAW 264.7 cell samples across time points. PC1 = 59.4% and PC2 = 16.7% of total variance (76.1% cumulative) indicate broad, coordinated shifts during infection. Per-sample PCA scores and loadings are provided in Supplementary S4 Table.

**Fig 2:**
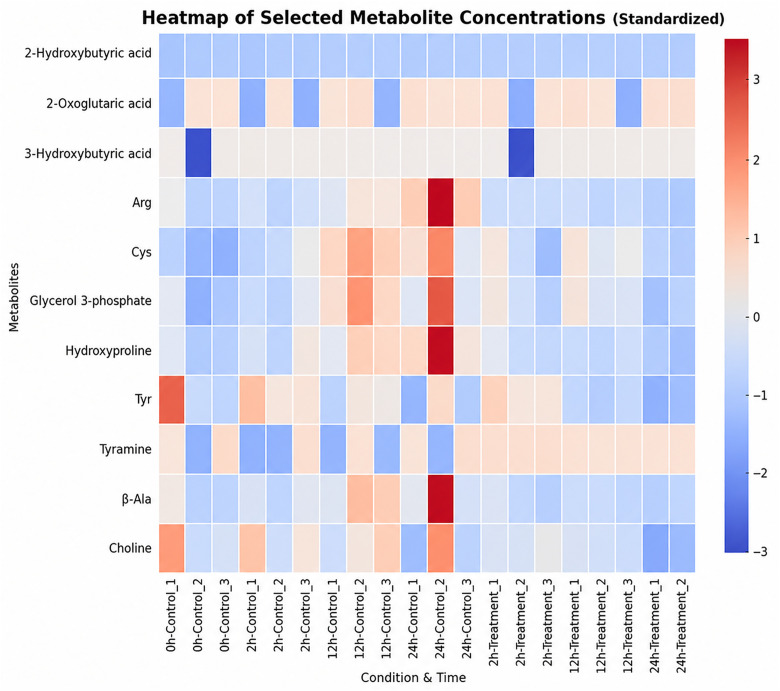
Heatmap of row-wise z-scored metabolites across all 21 samples. Row-wise z-score heatmap across 21 samples illustrates concordant replicate-level patterns and a temporal divergence consistent with univariate results. Hierarchical clustering groups samples by time point and condition. Underlying matrices and all row/column annotations are provided in Supplementary S5 Table. Numerical annotations were removed to improve visualization of relative pattern differences across samples and conditions.

PCA demonstrated separation between infected and control samples across time points (PC1 = 59.4%, PC2 = 16.7%; 76.1% cumulative), indicating broad, coordinated shifts; complete per-sample scores and explained variance are provided in Supplementary S4 Table. A heatmap based on row-wise z-scores across all 21 samples showed concordant replicate-level patterns consistent with the univariate results (Supplementary S5 Table; Figs 1 and 2).

**Fig 3:**
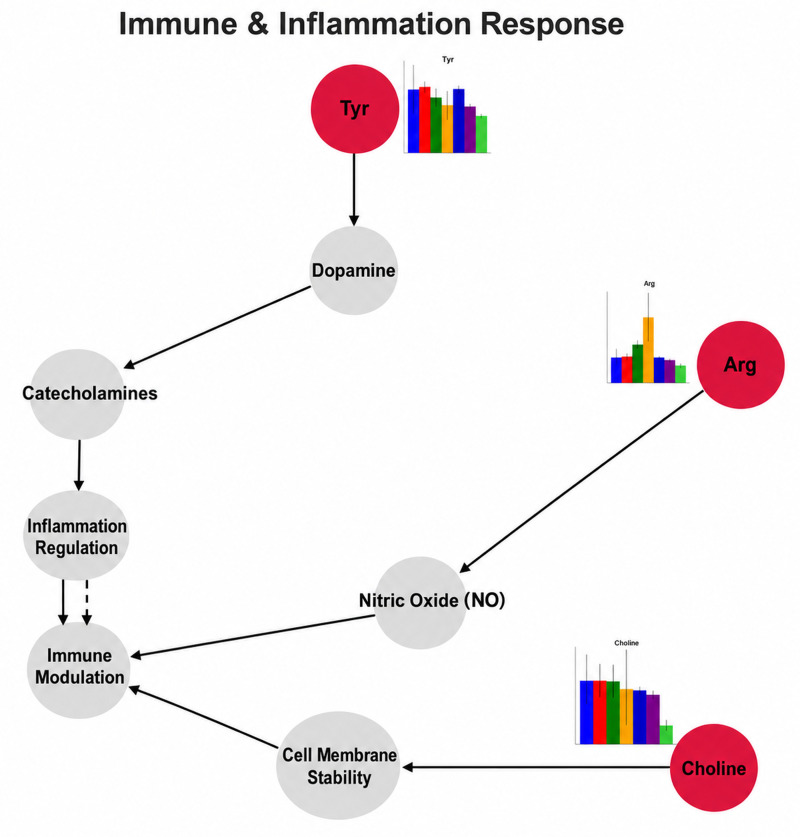
Metabolic pathway alterations in immune & inflammation response. Metabolites linked to macrophage immune biology—e.g., arginine (NO precursor) and tyrosine (catecholamine precursor)—were lower in infected cells relative to controls, most prominently at 24 h. These trends are presented as descriptive associations; cytokines were not directly measured. Numerical values for all replicates and group summaries are provided in Supplementary S3 Table. Bars represent mean metabolite abundance (pmol/10^6^ cells) from three biological replicates. Error bars indicate standard deviation. Inset bar plots show mean metabolite abundance across experimental groups and time points. Bars are presented in chronological order: 0h-Control, 2h-Control, 12h-Control, 24h-Control, 2h-Treatment, 12h-Treatment, and 24h-Treatment.

Consistent with immune‑associated pathways, arginine (a nitric oxide precursor) decreased (T/C ~ 0.25) and tyrosine showed a modest decrease (T/C ~ 0.68). These observations are descriptively consistent with metabolite alterations previously associated with NO-related and catecholamine-related pathways in macrophages. (Fig 3)

**Fig 4:**
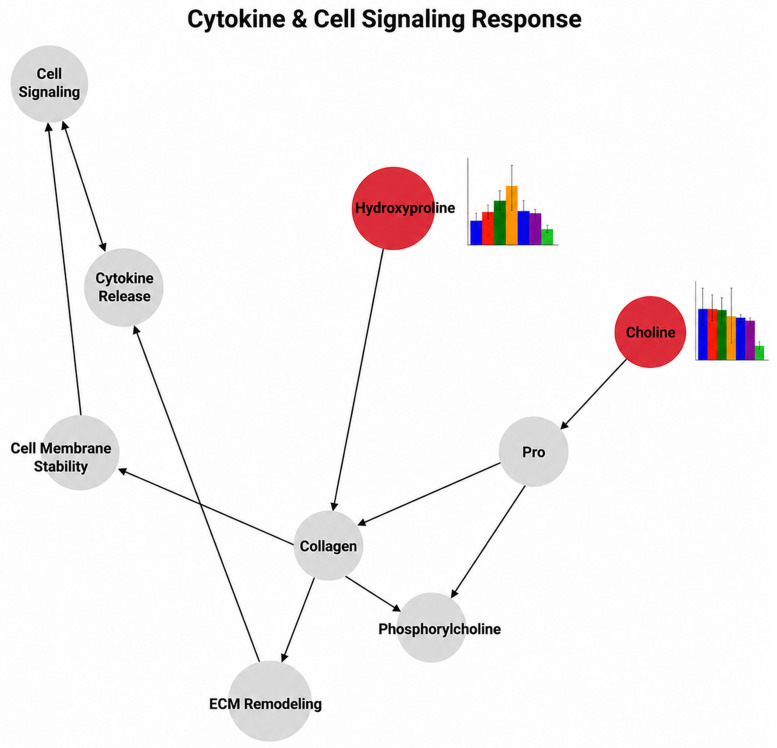
ECM/cell-signaling–associated metabolites. Hydroxyproline (collagen turnover surrogate) and choline (membrane-lipid/signaling) decreased in infected cells, consistent with metabolites previously associated with extracellular-matrix and membrane-lipid biology. These plots are descriptive; no matrix proteins or cytokines were directly measured. Numerical values are provided in Supplementary S3 Table. Bars represent mean metabolite abundance (pmol/10^6^ cells) from three biological replicates. Error bars indicate standard deviation. Inset bar plots show mean metabolite abundance across experimental groups and time points. Bars are presented in chronological order: 0h-Control, 2h-Control, 12h-Control, 24h-Control, 2h-Treatment, 12h-Treatment, and 24h-Treatment.

Metabolites linked to extracellular matrix and membrane‑lipid biology also trended lower. Hydroxyproline (collagen turnover) decreased (T/C ~ 0.24), and choline (phosphatidylcholine/lipid signaling) decreased (T/C ~ 0.30). These observations are descriptive and should not be interpreted as direct evidence of functional changes in extracellular-matrix remodeling or immune signaling. (Fig 4)

**Fig 5:**
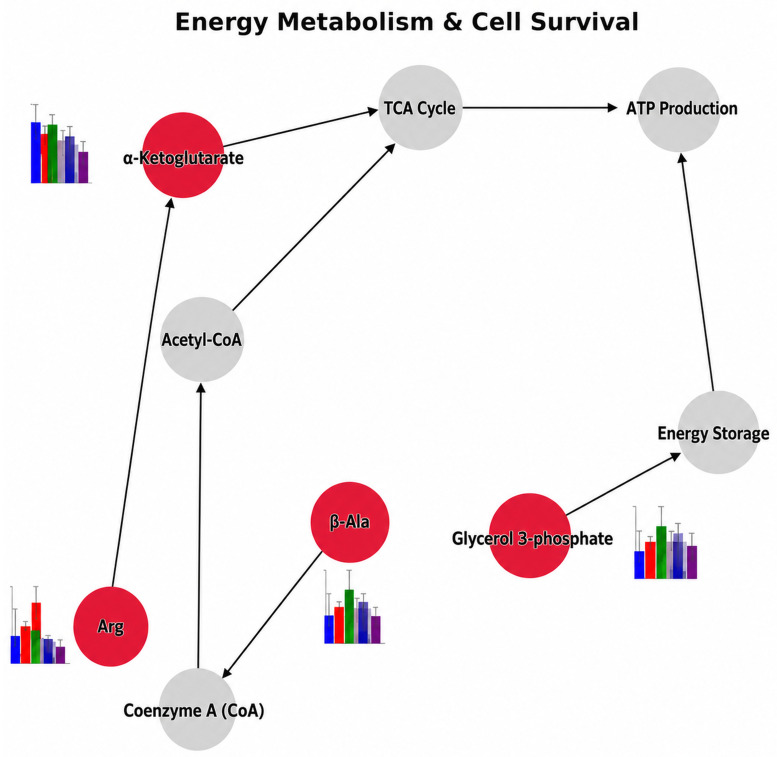
Energy metabolism and mitochondrial dysfunction pathway changes. Energy-linked metabolites including β-alanine and glycerol-3-phosphate decreased in infected cells, aligning with pathways connected to CoA synthesis and glycerolipid/energy metabolism. α-Ketoglutarate had insufficient 24 h replicate coverage; a lower mean was observed at 12 h and should be interpreted cautiously. Bars represent mean metabolite abundance (pmol/10^6^ cells) from three biological replicates. Error bars indicate standard deviation. Numerical values are provided in Supplementary S3 Table; for α-ketoglutarate, see the 12 h trend in Supplementary S3 Table. Inset bar plots show mean metabolite abundance across experimental groups and time points. Bars are presented in chronological order: 0h-Control, 2h-Control, 12h-Control, 24h-Control, 2h-Treatment, 12h-Treatment, and 24h-Treatment.

Energy‑related metabolites showed consistent decreases: β‑alanine (coenzyme A synthesis) at ~0.40 and glycerol‑3‑phosphate at ~0.47. For α‑ketoglutarate (TCA cycle), replicate coverage at 24 h was insufficient; a decrease was noted at 12 h (ratio ≈0.67) and should be interpreted cautiously. (Fig 5)

**Fig 6:**
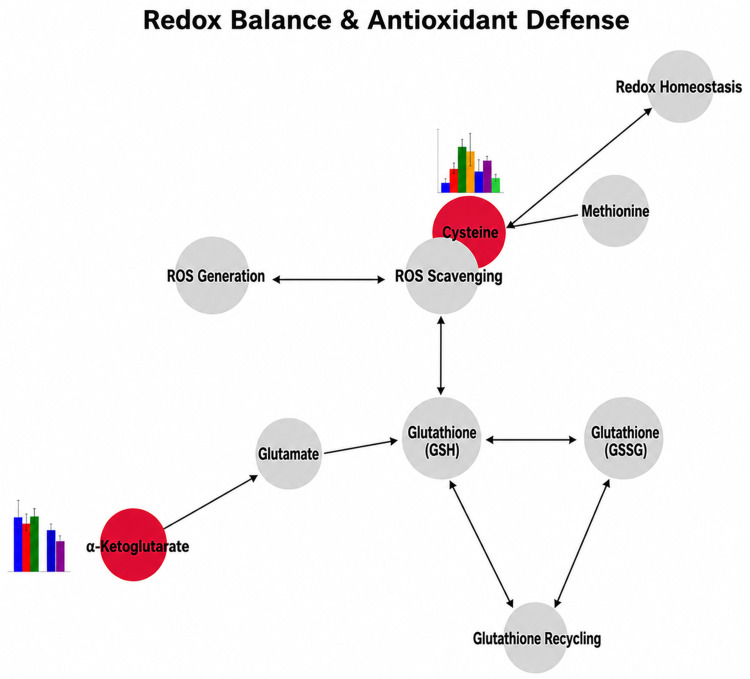
Redox balance and antioxidant defense alterations. Cysteine (a glutathione precursor) decreased in infected cells, consistent with adjustments in redox/glutathione-related metabolism. These data are descriptive; glutathione and oxidative-stress markers were not directly measured. Numerical values are provided in Supplementary S3 Table. Bars represent mean metabolite abundance (pmol/10^6^ cells) from three biological replicates. Error bars indicate standard deviation. Inset bar plots show mean metabolite abundance across experimental groups and time points. Bars are presented in chronological order: 0h-Control, 2h-Control, 12h-Control, 24h-Control, 2h-Treatment, 12h-Treatment, and 24h-Treatment.

Redox‑related cysteine (a glutathione precursor) decreased (T/C ~ 0.32), a pattern consistent with altered antioxidant capacity; we did not directly quantify glutathione or oxidative‑stress readouts. (Fig 6)

**Fig 7:**
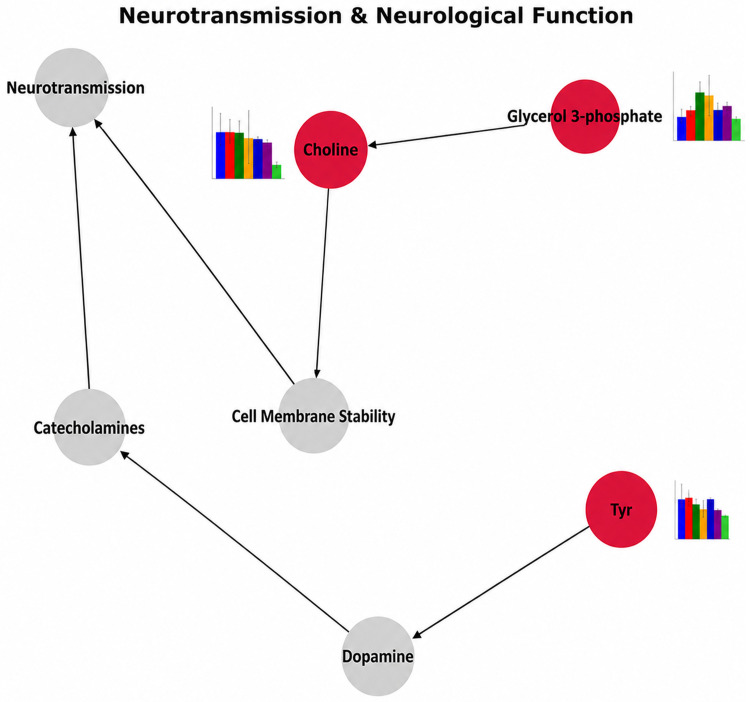
Metabolites associated with neuroimmune-related pathways. Metabolites with links to neurotransmitter biosynthesis/signaling showed downward trends at 24 h in infected cells. These plots are descriptive and do not constitute direct measurements of neurotransmitters or receptor activity. Numerical values are provided in Supplementary S3 Table. Bars represent mean metabolite abundance (pmol/10^6^ cells) from three biological replicates. Error bars indicate standard deviation. Inset bar plots show mean metabolite abundance across experimental groups and time points. Bars are presented in chronological order: 0h-Control, 2h-Control, 12h-Control, 24h-Control, 2h-Treatment, 12h-Treatment, and 24h-Treatment.

Metabolites with potential neuroimmune relevance, including tyrosine and choline, showed decreases compatible with broader neuroimmune interactions; we make no claims about neurotransmitter levels or signaling in the absence of direct measurements. (Fig 7)

Because infection efficiency and macrophage viability were not directly quantified, we additionally included an exploratory sensitivity analysis (Minimum Cell Loss, MCL) as an indirect assessment of whether large cell-number differences alone could plausibly account for the observed reductions. This analysis was intended only as supportive context and does not replace direct measurements of infection efficiency or cell viability. Detailed MCL estimates are provided in Supplementary S6 Table.

**Fig 8:**
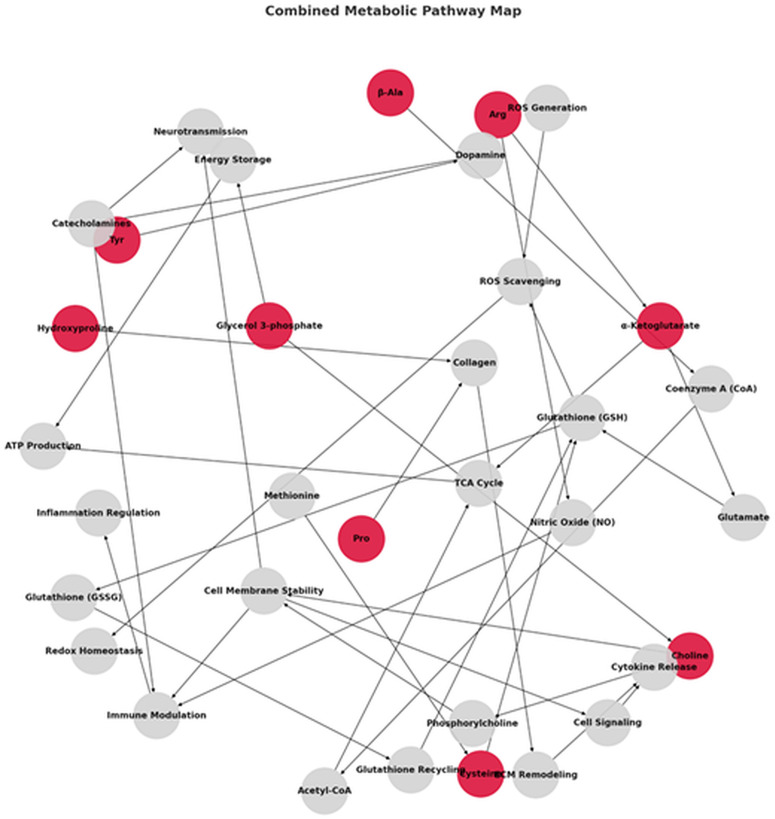
Integrated metabolic pathway map summarizing metabolic alterations. Schematic summarizes infection-associated changes across nitric-oxide–related, mitochondrial/energy, redox/glutathione, extracellular-matrix, membrane-lipid, and neuroimmune-related axes, highlighting the time-ordered nature of the shifts (most prominent at 24 h). All source values used for mapping trace to Table 1 and Supplementary S3 Table. The schematic is intended as a descriptive and hypothesis-generating summary and does not provide additional statistical inference.

Finally, we assembled an integrated pathway schematic to visually summarize the observed associations (Fig 8). The map depicts interconnections among changes spanning arginine/NO‑related, mitochondrial/energy, redox/glutathione, extracellular‑matrix, membrane‑lipid, and neuroimmune‑related metabolism; it is intended as a hypothesis‑generating, descriptive overview and does not add further statistical inference beyond the analyses reported above.

## Discussion

This study used targeted capillary electrophoresis–mass spectrometry (CE–MS) to profile time-dependent metabolic changes in RAW 264.7 cells infected with *B. canis*. The largest differences appeared at 24 h, with pronounced decreases in arginine, hydroxyproline, choline and cysteine (treatment/control ≈ 0.24–0.32), alongside β-alanine and glycerol-3-phosphate (≈ 0.40 and ≈ 0.47) and a modest change in tyrosine (≈ 0.68). Unsupervised analyses supported group-level separation over time (PCA PC1 = 59.4%, PC2 = 16.7%; 76.1% cumulative), with heatmaps showing concordant replicate-level trends. Formal testing used Welch’s t-tests with Benjamini–Hochberg false-discovery-rate control (full p and q in Supplementary S3 Table), and our interpretation emphasizes effect sizes and the time-ordered coherence of the responses. Given the exploratory nature of this targeted metabolomics analysis and the limited sample size (n = 3 per group), we emphasize effect sizes and consistent temporal trends rather than relying solely on statistical significance. Because macrophage biomass substantially exceeded bacterial biomass under the experimental conditions, the measured metabolite profiles were interpreted primarily as host-associated, although some contribution from bacterial metabolites cannot be excluded. We additionally report an exploratory upper-bound sensitivity estimate (Minimum Cell Loss) as contextual information regarding potential cell-number effects. These observations provide an association-level baseline and motivate targeted validation of the implicated pathways.

A major observation in our dataset was the decrease in arginine at 24 h (treatment/control ≈ 0.25). Arginine is widely recognized as a metabolite associated with macrophage nitric-oxide–related pathways and immunometabolic regulation [6,11]. In the context of macrophage biology, arginine sits at the intersection of iNOS-dependent NO production and arginase-dependent ornithine/polyamine synthesis; shifts in this balance can influence antimicrobial effector responses and immunomodulatory tone [6,11]. Although we did not directly quantify NO, iNOS expression, or cytokine outputs, the array-level behavior of arginine aligns with literature in *Brucella* and other intracellular pathogens reporting infection-associated adjustments of arginine handling [26–28]. Per-timepoint Welch’s tests with Benjamini–Hochberg FDR did not yield findings at q < 0.05 at this sample size (see S3 Table), so we interpret these effects as associations rather than definitive impairments. Importantly, our exploratory sensitivity analysis (Minimum Cell Loss) suggested that substantial cell-number differences would be required to fully account for the largest observed decreases, although direct measurements of infection efficiency and viability were not performed. Collectively, these observations are compatible with altered arginine/NO-related pathways during *B. canis* infection and justify targeted follow-up to quantify infection kinetics, iNOS/NO production, and cationic amino-acid transporter activity in the same system.

We also noted a modest decrease in tyrosine (treatment/control ≈ 0.68). Tyrosine is widely recognized as a precursor associated with catecholamine-related pathways and broader neuroimmune regulatory processes [29–32]. Previous studies have described interactions between amino-acid metabolism, catecholamine-associated signaling, and immune regulation in macrophages and other immune systems [29–32]. In addition, choline metabolism has been linked to membrane-lipid organization and signaling-related pathways in prior studies [33,34]. However, neurotransmitters, receptor activity, signaling pathways, and downstream immune responses were not directly measured in the present study. Therefore, the observed decreases in tyrosine and choline should be interpreted only as descriptive metabolite-level observations rather than direct evidence of functional neuroimmune or signaling alterations. As with arginine, the lack of q < 0.05 after BH-FDR at this sample size further supports a conservative interpretation of these findings.

Energy-linked metabolites exhibited coordinated decreases, notably β-alanine and glycerol-3-phosphate, in a manner consistent with impacts on coenzyme-A biosynthesis and glycerolipid/energy metabolism [35]. Such shifts are conceptually aligned with the heightened energetic and biosynthetic demands that accompany intracellular infection, during which macrophages adjust mitochondrial substrate handling, anaplerosis, and lipid remodeling [36,37]. For α-ketoglutarate, 24 h data were not reported because replicate coverage was insufficient; a lower mean was observed at 12 h and should be interpreted cautiously in light of the same statistical considerations. The aggregate pattern therefore suggests infection-associated adjustments of TCA-linked and lipid-linked nodes described in macrophages and other intracellular pathogen models [11,12], while remaining descriptive given our reliance on targeted profiling and absence of direct mitochondrial flux measurements. Together with the MCL upper-bound estimates, these findings argue for follow-up studies that pair metabolite quantification with flux tracing and high-content viability/infection-rate readouts, enabling clearer separation of true pathway modulation from cell-number effects.

Cysteine, a precursor for glutathione, showed a pronounced decrease, consistent with adjustments in redox/glutathione metabolism under intracellular infection stress [16,38]. Given cysteine’s centrality to glutathione synthesis and thiol-dependent antioxidant defenses, even association-level decreases raise the possibility of altered redox buffering capacity in infected macrophages. We did not quantify glutathione pools, oxidative-stress markers, or NADPH dynamics in this study; accordingly, we avoid causal phrasing and view these results as hypothesis-generating. Targeted assays—total/oxidized glutathione, ROS readouts, and enzyme activities—combined with infection-rate controls [16,21] would directly test whether the cysteine trend maps onto measurable changes in redox homeostasis or reflects broader shifts in amino-acid availability accompanying infection.

Hydroxyproline and choline also decreased at 24 h. Hydroxyproline is commonly associated with collagen-related metabolism and extracellular-matrix biology [39], whereas choline contributes to phosphatidylcholine and broader membrane-lipid metabolism [39,40]. Previous studies have linked these metabolites to processes involving matrix organization and membrane-associated cellular functions [39,40]. However, matrix proteins, lipid classes, receptor localization, and signaling activity were not directly measured in the present study. Therefore, these observations should be interpreted as descriptive metabolite-level associations rather than direct evidence of functional extracellular-matrix remodeling or membrane-signaling alterations.

Finally, the decrease in glycerol-3-phosphate—considering its roles at the interface of glycolysis, glycerolipid synthesis, and redox shuttling—remains compatible with adjustments in lipid/energy pathways that can influence membrane composition and downstream signaling [36,37]. Similar infection-associated lipid and energy-metabolism trends have been documented in macrophages and other intracellular systems [11,17], and our findings align with those reports at an association level. Because our study did not include lipidomics or flux-based measurements, we avoid mechanistic claims; instead, we propose that future work prioritize isotope-tracing and quantitative lipid profiling under matched MOI/viability controls to determine whether the glycerol-3-phosphate trend reflects pathway-level modulation versus secondary consequences of infection dynamics. Within these boundaries, the present data provide a transparent baseline for hypothesis testing around glycerolipid metabolism in *B. canis*–infected macrophages.

This study has several limitations. First, intracellular bacterial burden, infection efficiency, and macrophage viability were not directly quantified at each sampling time point. Therefore, the metabolite alterations reported here should be interpreted cautiously, as contributions from differences in infection dynamics, bacterial burden, or host cell status cannot be fully excluded.

Because infection status was not directly characterized at each sampling time point, the observed metabolic responses cannot be attributed specifically to successful intracellular replication of *B. canis*. The measured profiles may reflect a combination of bacterial attachment, host–pathogen interaction, and intracellular infection processes [7,20]. Previous studies have shown that Brucella–macrophage interactions encompass multiple stages, including bacterial attachment, intracellular uptake, persistence, and replication, and that productive intracellular infection depends on both bacterial and host physiological conditions [7,20]. Therefore, the relative contribution of these different interaction states could not be distinguished in the present study. Accordingly, the present findings should be interpreted as metabolic responses associated with *B. canis* exposure rather than as specific signatures of intracellular replication.

Second, because of the exploratory design, limited sample size (n = 3 per group), and absence of direct functional assays, the present findings should be regarded as descriptive and hypothesis-generating rather than definitive evidence of pathway-level functional changes. Third, the use of a single macrophage cell line limits broader biological interpretation, and additional studies using complementary macrophage models, direct infection-efficiency measurements, viability assays, and pathway-specific functional readouts will be required to validate the observed metabolite patterns.

## Conclusion

This study provides a preliminary time-resolved metabolomic profile of RAW 264.7 macrophages following *B. canis* infection. Several metabolites associated with amino-acid, redox, lipid, and energy metabolism showed coordinated decreases, most prominently at 24 h post-infection.

Beacuse of no direct quantification of infection efficiency and macrophage viability, exploratory design and limited sample size, these findings should be interpreted cautiously. Nevertheless, the observed temporal metabolite patterns provide a descriptive framework that may help guide future studies incorporating direct measurements of infection efficiency, viability, and pathway-specific functional validation.

## Supporting information

S1 Table Sample metadata. Study-wide sample information, including sample IDs, condition (Infected/Control), time point (e.g., 0 h, 12 h, 24 h), replicate labels, and QC flags used in downstream analyses.
(XLSX)

S2 Table Targeted metabolite reference/annotation list. Compound identifiers and reference metadata used for targeted assignment: metabolite name, formula, reference IDs (e.g., KEGG/HMDB), m/z (theoretical and measured), mass error (ppm), migration time (MT), adduct, quantitation/annotation status, and notes on potential isomers where applicable.
(XLSX)

S3 Table Integrated dataset. Single consolidated file containing: (i) replicate-level quantified values (pmol/10^6^ cells) for all samples; (ii) per–time point summary statistics from Welch’s t-tests with Benjamini–Hochberg FDR (reporting p and q), including group means, SD, n, treatment/control ratio (T/C), and log2FC; and (iii) the Minimum Cell Loss (MCL) sensitivity analysis. Note: α-ketoglutarate at 24 h is not reported due to insufficient replicate coverage; a 12 h trend is provided within the file.
(XLSX)

S4 Table PCA scores and explained variance. Per-sample principal-component scores and loadings, eigenvalues, and variance explained (including cumulative percentages) corresponding to Fig 1.
(XLSX)

S5 Table Heatmap matrices and annotations. Row-wise z-scored matrix used to generate Fig 2, with complete row/column annotations (pathway/role labels; time point and condition per sample) and clustering parameters.
(XLSX)

S6 Table MCL sensitivity from replicate-level values. Per-metabolite Minimum Cell Loss (MCL) estimates computed directly from replicate-level values, providing the minimum fraction of cell-number difference required to fully account for the observed treatment/control ratios. Includes input summaries (group means, SD, *n*), computed MCL values, and notes on interpretation. General notes for all supplementary files. Concentrations are reported as pmol/10^6^ cells. Missing or QC-excluded entries are indicated as NA/flagged. Numerical values underlying Figs 3–7 are included in S3 Table; PCA score tables are in S4 Table; heatmap matrices and annotations are in S5 Table.
(XLSX)
