# Supplementary material for: Targeted metabolomic profiling of RAW 264.7 cells infected with Brucella canis identifies time-dependent metabolic changes
Source: PLoS One. 2026 Jul 16;21(7):e0353470. doi: 10.1371/journal.pone.0353470 (PMC13375135; doi:10.1371/journal.pone.0353470)
Supplement: Attachment — Submitted filename: Response to Reviewers 0512 (1).pdf [file pone.0353470.s008.pdf]

Response to Reviewers

Manuscript title: Targeted metabolomic profiling of RAW 264.7 cells infected with *Brucella canis* identifies time-dependent metabolic changes

Manuscript ID: PONE-D-26-09735

Dear Editor,

We sincerely thank the Editor and Reviewers for their careful evaluation and constructive comments. We greatly appreciate the thoughtful suggestions, which substantially improved the clarity, rigor, and interpretation of the manuscript. In response, we extensively revised the manuscript to address the concerns raised. All changes are highlighted in red in the revised manuscript.

Reviewer #1

Comment:

The authors have not provided information regarding macrophage viability or infection efficiency. The MCL calculation is not convincing. Without CFU and viability information, the data are not trustworthy.

Response:

We thank the reviewer for this important comment. In the revised manuscript, we further moderated the interpretation of the Minimum Cell Loss (MCL) analysis and clarified that it is presented only as exploratory contextual information rather than as a substitute for direct infection-efficiency or viability measurements.

In addition, the limitation paragraph and conclusion section were revised to further emphasize the descriptive and hypothesis-generating nature of the present metabolomics analysis.

These revisions were implemented in the Results section (Page 11, Lines 235-240), Limitation section (Page 16, Lines 341-351), and Conclusion section (Page 16, Lines 354-362), highlighted in the revised manuscript.

Comment:

The conclusions regarding pathways and cytokine secretion remain speculative.

Response:

We agree with the reviewer that some interpretations in the original manuscript were overly speculative. In the revised manuscript, statements implying direct effects on cytokine secretion, extracellular-matrix remodeling, neurotransmission, oxidative stress, or pathway activation were revised or removed unless directly supported by the experimental data.

The manuscript now consistently emphasizes that the findings represent descriptive metabolite-level associations and hypothesis-generating observations rather than definitive mechanistic conclusions.

These revisions were implemented in the Results section (Page 10, Lines 217-219) and Discussion section (Page 13, Lines 284-295 and Page 15, Lines 320-328), highlighted in the revised manuscript.

Comment:

Lines 97–100: The authors state that the findings help differentiate between *B. canis* and other *Brucella* species; however, no information is provided to support this claim.

Response:

We thank the reviewer for this helpful comment. The Introduction section was revised to remove unsupported comparative claims regarding differentiation from other *Brucella* species. The revised text now more conservatively states that the present study may provide a preliminary framework for future comparative investigations.

This revision was implemented in the Introduction section (Page 5, Lines 97–100), highlighted in the revised manuscript.

Comment:

The legend of Fig. 1 is oddly organized.

Response:

We thank the reviewer for this helpful suggestion. The Figure 1 legend was reorganized to improve the sequential ordering of experimental conditions and time points and enhance readability.

This revision was implemented in Figure 1 and its legend, highlighted in the revised manuscript.

Reviewer #2

Comment:

A major concern was the lack of experimental data regarding intracellular trafficking and infection status at the timepoints of sampling.

Response:

We appreciate this important comment. As suggested, the revised manuscript now explicitly acknowledges that intracellular bacterial burden, infection efficiency, and macrophage viability were not directly quantified during the experiment. We also clarified that the MCL analysis was included only as exploratory contextual information and does not replace direct infection-status measurements.

These revisions were implemented in the Results section (Page 11, Lines 235-240), Limitation paragraph (Page 16, Lines 341-351), and Conclusion section (Page 16, Lines 354-362), highlighted in the revised manuscript.

Comment:

Figure 2. I would eliminate numerical data on the heat map.

Response:

We thank the reviewer for this valuable suggestion. To improve readability and facilitate visual comparison across conditions and replicates, numerical annotations were removed from the heatmap in Figure 2 while preserving the original clustering structure and color scaling.

This revision was implemented in Figure 2 and its legend (Page 25, Lines 504-505), highlighted in the revised manuscript.

Comment:

Figure 3–7. The bar graphs are not clear what they represent and the figure legends do not give clarity on this.

Response:

We appreciate this helpful suggestion. The legends for Figures 3–7 were revised to clarify what the plotted bars represent, including explicit descriptions of mean metabolite abundance, biological replicates, and standard deviation error bars.

These revisions were implemented in the Figure Legends section (Pages 25–26, Lines 511-538), highlighted in the revised manuscript.

We believe that these revisions have substantially improved the clarity, transparency, and scientific rigor of the manuscript. We sincerely thank the reviewers for their constructive comments and hope that the revised manuscript is now suitable for reconsideration.
