# Supplementary material for: Targeted metabolomic profiling of RAW 264.7 cells infected with Brucella canis identifies time-dependent metabolic changes
Source: PLoS One. 2026 Jul 16;21(7):e0353470. doi: 10.1371/journal.pone.0353470 (PMC13375135; doi:10.1371/journal.pone.0353470)
Supplement: Attachment — Submitted filename: Responses to Reviewers -PONE-D-26-09735R1.pdf [file pone.0353470.s009.pdf]

**Response to Reviewers**

Manuscript title: Targeted metabolomic profiling of RAW 264.7 cells infected with *Brucella canis* identifies time-dependent metabolic changes

Manuscript ID: PONE-D-26-09735R1

Dear Editor,

We sincerely thank the Editor and Reviewers for their careful evaluation and constructive comments. We greatly appreciate the thoughtful suggestions, which substantially improved the clarity, rigor, and interpretation of the manuscript. In response, we extensively revised the manuscript to address the concerns raised. All changes are highlighted in red in the revised manuscript.

**Reviewer #2**

**Comment:**

The authors should more clearly emphasize that infection outcome was not determined at each metabolic sampling time point and that the observed responses may reflect bacterial attachment, host–pathogen interaction, intracellular infection, or a combination of these processes.

**Response:**

We thank the reviewer for this insightful comment. We agree that, because infection status was not directly characterized at each sampling time point, the observed metabolic responses cannot be attributed specifically to successful intracellular replication of *B. canis*. In response, we expanded the limitation section to provide additional biological context regarding the interpretation of the observed metabolite profiles. Specifically, we clarified that Brucella–macrophage interactions may encompass multiple stages, including bacterial attachment, intracellular uptake, persistence, and replication, and that productive intracellular infection depends on both bacterial and host physiological conditions [7, 20]. Therefore, the measured metabolite profiles may reflect a combination of bacterial attachment, host–pathogen interaction, and intracellular infection processes, and the relative contribution of these interaction states could not be distinguished in the present study. We further clarified that the findings should be interpreted as metabolic responses associated with *B. canis* exposure rather than as specific signatures of intracellular replication.

This revision was implemented in the Limitation section (Page 15-16, Lines 338–347), highlighted in the revised manuscript.

**Comment:**

Regarding Figures 3–7, the meaning of the inset bar plots and their color coding is not sufficiently clear.

**Response:**

We appreciate this helpful suggestion. The legends of Figures 3–7 were revised to clarify the interpretation of the inset bar plots. Specifically, we added descriptions indicating that the inset bar plots represent mean metabolite abundance across experimental groups and time points and clarified the chronological order of the displayed groups (0h-Control, 2h-Control, 12h-Control, 24h-Control, 2h-Treatment, 12h-Treatment, and 24h-Treatment).

These revisions were implemented in the Figure Legends section (Pages 25–27), highlighted in the revised manuscript.

We believe that these revisions have substantially improved the clarity, transparency, and scientific rigor of the manuscript. We sincerely thank the reviewers for their constructive comments and hope that the revised manuscript is now suitable for reconsideration.
