# Supplementary material for: Targeted metabolomic profiling of RAW 264.7 cells infected with Brucella canis identifies time-dependent metabolic changes
Source: PLoS One. 2026 Jul 16;21(7):e0353470. doi: 10.1371/journal.pone.0353470 (PMC13375135; doi:10.1371/journal.pone.0353470)
Supplement: Attachment — Submitted filename: Response to Reviewers 0312.docx [file pone.0353470.s007.docx]

**Response to Reviewers**

Manuscript title: Targeted metabolomic profiling of RAW 264.7 cells infected with *Brucella canis* identifies time-dependent metabolic changes

Previous manuscript ID: PONE-D-25-39090

Dear Editor,

We would like to thank the editor and reviewers for their careful evaluation and the constructive comments of our previous submission to your journal, PLoS One. We have carefully read the comments and revised the manuscript to address the reviewers’ concerns and to improve the clarity, rigor, and transparency of the study.

Based on the comments, we have comprehensively revised the Abstract, Results and Discussion sections of our manuscript, reflecting the comments from editor and reviewers. General revision sections of our manuscript are highlighted in green. We have also revised the paper to specifically reflect the comments from reviewers, and these sections are highlighted in yellow. The lines were mentioned in the detailed point-by-point responses.

Importantly, we revised the manuscript title to better reflect the descriptive and hypothesis-generating nature of the metabolomics analysis and to avoid overinterpretation of the findings.

In addition, we substantially improved data transparency and reproducibility by providing the full metabolomics dataset and associated analysis outputs as supplementary datasets (S1–S6), including sample metadata, annotation lists, integrated metabolomics data, PCA scores, heatmap matrices, and a sensitivity analysis evaluating the potential impact of cell number differences.

Detailed point-by-point responses to the reviewers’ comments are provided below.

**Reviewer #1**

Comment:

The authors employ a metabolomic approach to analyze metabolic perturbations during *Brucella canis* infection in macrophages. However, additional information regarding macrophage infection such as fluorescence of infection at different timepoints or colony forming units (CFU) recovered should be included to better understand infection dynamics.

Response:

We thank the reviewer for this important comment. In the revised manuscript, we have clarified the infection procedures and explicitly acknowledged this limitation. Specifically, the Materials and Methods section now states that although cell morphology and viability were visually confirmed prior to infection, quantitative infection efficiency measurements such as CFU enumeration or fluorescence-based infection assays were not performed (Lines 118–121 in page 6, Cell culture and infection section; highlighted with yellow color in the revised manuscript).

Because the present study was designed as an exploratory metabolomics analysis focusing on time-dependent metabolic responses, our experimental design prioritized standardized infection conditions and metabolite profiling across multiple time points. Also, we thought we did not need those experiments even though there is a possibility that the infected bacteria may not infect cells evenly. Therefore, detailed infection-kinetics assays such as CFU enumeration or fluorescence-based infection assays were not carried out in the present experiment. This limitation is now explicitly acknowledged in the revised manuscript (Lines 358-361 in page 16: highlighted with yellow color in the revised manuscript).

Furthermore, we have moderated the interpretation of the metabolomic findings to avoid over-interpretation and to emphasize that the observed metabolic changes represent association-level observations rather than definitive mechanistic conclusions. These revisions were implemented in the Results and Discussion sections (Lines 211–214 in page 10, 230–237 in page 11, and Lines 254–263 in page 12; highlighted with yellow color in the revised manuscript).

**Reviewer #2**

Comment:

There are concerns regarding the experimental design, particularly the lack of discussion regarding macrophage viability and effective colonization of the macrophages by *B. canis*.

Response:

We appreciate this valuable comment and agree that infection efficiency and macrophage viability are important considerations when interpreting metabolomic data.

As described above in response to Reviewer #1, the revised manuscript now explicitly acknowledges this limitation in the Materials and Methods section (Page 6, Lines 118–121; highlighted with yellow color in the revised manuscript).

In addition, to provide further context for interpreting the metabolite decreases observed in infected cells, we introduced a sensitivity analysis termed Minimum Cell Loss (MCL). This analysis estimates the theoretical upper bound of cell-number differences required to account for the observed metabolite reductions. The description of this analysis has been added to the Statistical Analysis section (Lines 173–185 in pages 8 ~ 9; highlighted with yellow color in the revised manuscript), and the corresponding results are provided in Supplementary Dataset S6.

This analysis indicates that very large differences in cell numbers (approximately 53–76%) would be required to fully explain the largest metabolite reductions observed at 24 hours. However, because infection efficiency and cell viability were not directly measured, these estimates are presented only as contextual information.

These additions provide additional context for interpreting the metabolomics results while acknowledging the limitations of the current experimental design.

Comment:

The conclusions regarding pathways and cytokine secretion appear speculative based on limited metabolite changes.

Response:

We agree with the reviewer that some interpretations in the original manuscript may have been overly speculative.

In the revised manuscript, the relevant sections have been carefully revised to ensure that interpretations remain descriptive and appropriately supported by the metabolomic data. Statements implying direct effects on cytokine secretion, immune signaling, or specific biological pathways have been removed or reframed as association-level observations.

These revisions were implemented in the Results section (Lines 211–214 in page 10 and Lines 230–237 in page 11; highlighted with yellow color in the revised manuscript) and in the Discussion section (Lines 252–263 in page 12; highlighted with yellow color in the revised manuscript).

The manuscript now clearly emphasizes that the findings provide a hypothesis-generating framework for future mechanistic studies rather than definitive conclusions regarding pathway regulation.

Comment:

The legend of Fig. 1 is oddly organized.

Response:

The figure legend has been revised and reorganized to improve clarity and readability.

Comment:

There is no mention about how to access the raw metabolomics data.

Response:

We thank the reviewer for this important suggestion. To improve transparency and reproducibility, we have substantially expanded the availability of the underlying metabolomics data.

All datasets used in this study are now provided as supplementary files accompanying the manuscript:

S1 Dataset: Sample metadata
S2 Dataset: Targeted metabolite reference and annotation list
S3 Dataset: Integrated metabolomics dataset containing replicate-level quantified values and statistical outputs
S4 Dataset: PCA scores and explained variance
S5 Dataset: Heatmap matrices and annotations
S6 Dataset: Minimum Cell Loss (MCL) sensitivity analysis

These supplementary files provide the complete metabolomics measurements, statistical outputs, and analysis matrices used in the study, allowing full transparency and reproducibility of the analyses (Data Availability section, Page 18; highlighted with yellow color in the revised manuscript).

We believe that these revisions have substantially improved the clarity, transparency, and scientific rigor of the manuscript. We sincerely thank the reviewers for their constructive comments and hope that the revised manuscript is now suitable for reconsideration.
