# Supplementary material for: Targeted metabolomic profiling of RAW 264.7 cells infected with Brucella canis identifies time-dependent metabolic changes
Source: PLoS One. 2026 Jul 16;21(7):e0353470. doi: 10.1371/journal.pone.0353470 (PMC13375135; doi:10.1371/journal.pone.0353470)
Supplement: Peer review history

# Author comment: Author response to Decision Letter 0

**DOI:** 10.1371/journal.pone.0353470.r001

13 Mar 2026

Attachment Submitted filename: Response to Reviewers 0312.docx

# Peer review: Decision Letter 0

**DOI:** 10.1371/journal.pone.0353470.r002

**Authors:** Roy Martin Roop II

30 Apr 2026

PONE-D-26-09735

Targeted metabolomic profiling of RAW 264.7 cells infected with  *Brucella canis*   identifies time-dependent metabolic changes

PLOS One

Dear Dr. Yoo,

Thank you for submitting your manuscript to PLOS ONE. After careful consideration, we feel that it has merit but does not fully meet PLOS ONE’s publication criteria as it currently stands. Therefore, we invite you to submit a revised version of the manuscript that addresses the points raised during the review process.

Please submit your revised manuscript by Jun 14 2026 11:59PM. If you will need more time than this to complete your revisions, please reply to this message or contact the journal office at plosone@plos.org. When you're ready to submit your revision, log on to https://www.editorialmanager.com/pone/ and select the 'Submissions Needing Revision' folder to locate your manuscript file.

Please include the following items when submitting your revised manuscript:

- A letter that responds to each point raised by the academic editor and reviewer(s). You should upload this letter as a separate file labeled 'Response to Reviewers'.
- A marked-up copy of your manuscript that highlights changes made to the original version. You should upload this as a separate file labeled 'Revised Manuscript with Track Changes'.
- An unmarked version of your revised paper without tracked changes. You should upload this as a separate file labeled 'Manuscript'.

If you would like to make changes to your financial disclosure, please include your updated statement in your cover letter. Guidelines for resubmitting your figure files are available below the reviewer comments at the end of this letter.

If applicable, we recommend that you deposit your laboratory protocols in protocols.io to enhance the reproducibility of your results. Protocols.io assigns your protocol its own identifier (DOI) so that it can be cited independently in the future. For instructions see: https://journals.plos.org/plosone/s/submission-guidelines#loc-laboratory-protocols. Additionally, PLOS ONE offers an option for publishing peer-reviewed Lab Protocol articles, which describe protocols hosted on protocols.io. Read more information on sharing protocols at https://plos.org/protocols?utm_medium=editorial-email&utm_source=authorletters&utm_campaign=protocols.

As the corresponding author, your ORCID iD is verified in the submission system and will appear in the published article. PLOS supports the use of ORCID, and we encourage all coauthors to register for an ORCID iD and use it as well. Please encourage your coauthors to verify their ORCID iD within the submission system before final acceptance, as unverified ORCID iDs will not appear in the published article. *Only*  the individual author can complete the verification step; PLOS staff *cannot*  verify ORCID iDs on behalf of authors.

We look forward to receiving your revised manuscript.

Kind regards,

R. Martin Roop II, Ph.D.

Academic Editor

*PLOS One*

Journal Requirements:

When submitting your revision, we need you to address these additional requirements.

1. Please ensure that your manuscript meets PLOS ONE's style requirements, including those for file naming. The PLOS ONE style templates can be found at

https://journals.plos.org/plosone/s/file?id=wjVg/PLOSOne_formatting_sample_main_body.pdf and

https://journals.plos.org/plosone/s/file?id=ba62/PLOSOne_formatting_sample_title_authors_affiliations.pdf

2. Please note that funding information should not appear in any section or other areas of your manuscript. We will only publish funding information present in the Funding Statement section of the online submission form. Please remove any funding-related text from the manuscript.

3. We note that the grant information you provided in the ‘Funding Information’ and ‘Financial Disclosure’ sections do not match.

When you resubmit, please ensure that you provide the correct grant numbers for the awards you received for your study in the ‘Funding Information’ section.

4. Thank you for stating the following financial disclosure:

“This study was performed with the support of the “Cooperative Research Program of Center for Companion Animal Research (Project No. PJ01398501)” Rural Development Administration, Republic of Korea and by the BK21 PLUS program and the Veterinary Research Institute of the College of Veterinary Medicine, Seoul National University, Korea.”

Please state what role the funders took in the study.  If the funders had no role, please state: "The funders had no role in study design, data collection and analysis, decision to publish, or preparation of the manuscript."

If this statement is not correct you must amend it as needed.

Please include this amended Role of Funder statement in your cover letter; we will change the online submission form on your behalf.

5. If the reviewer comments include a recommendation to cite specific previously published works, please review and evaluate these publications to determine whether they are relevant and should be cited. There is no requirement to cite these works unless the editor has indicated otherwise.

Additional Editor Comments:

As you can see from their comments below, I received two very different evaluations of your manuscript. Considering the serious concerns raised by Reviewer 1 I am going to offer you the opportunity to submit a revised version of  the paper that adequately and appropriately all of the concerns raised by both reviewers. But fully and clearly addressing the concerns raised by Reviewer 1 will be the major factor for my determination of whether the revised manuscript meets the criteria set for publication in the journal.

[Note: HTML markup is below. Please do not edit.]

Reviewers' comments:

Reviewer's Responses to Questions

**Comments to the Author**

1. Is the manuscript technically sound, and do the data support the conclusions?

The manuscript must describe a technically sound piece of scientific research with data that supports the conclusions. Experiments must have been conducted rigorously, with appropriate controls, replication, and sample sizes. The conclusions must be drawn appropriately based on the data presented.

Reviewer #1: No

Reviewer #2: Yes

**********

2. Has the statistical analysis been performed appropriately and rigorously?

Reviewer #1: No

Reviewer #2: Yes

**********

3. Have the authors made all data underlying the findings in their manuscript fully available?

The PLOS Data policy requires authors to make all data underlying the findings described in their manuscript fully available without restriction, with rare exception (please refer to the Data Availability Statement in the manuscript PDF file). The data should be provided as part of the manuscript or its supporting information, or deposited to a public repository. For example, in addition to summary statistics, the data points behind means, medians and variance measures should be available. If there are restrictions on publicly sharing data—e.g. participant privacy or use of data from a third party—those must be specified.

Reviewer #1: Yes

Reviewer #2: Yes

**********

4. Is the manuscript presented in an intelligible fashion and written in standard English?

PLOS ONE does not copyedit accepted manuscripts, so the language in submitted articles must be clear, correct, and unambiguous. Any typographical or grammatical errors should be corrected at revision, so please note any specific errors here.

Reviewer #1: Yes

Reviewer #2: Yes

**********

5. Review Comments to the Author

Please use the space provided to explain your answers to the questions above. You may also include additional comments for the author, including concerns about dual publication, research ethics, or publication ethics. (Please upload your review as an attachment if it exceeds 20,000 characters)

Reviewer #1: This manuscript describes a metabolomic analysis of Brucella canis-infected macrophages, and the authors indicate that the levels of several metabolites are significantly altered in infected macrophages compared to uninfected macrophages. In turn, physiological outcomes in the bacteria that result from these metabolic changes are predicted.

The experimental design is deeply flawed with significant concerns about the validity of the data, and more importantly, the data presented do not support the claims made by the authors.

Experimental design: How did the analysis differentiate between metabolites from the macrophages or the bacteria? This is likely not a major factor in the results, but it should be discussed. From the authors' response to the reviewers, it appears that the authors were previously pointed to the concern of a lack of discussion of macrophage viability or the effective colonization of the macrophages by B. canis. The authors have attempted to address this concern with the MCL calculation, but this approach is not convincing or appropriate. Lines 259-263 make a statement but provide no meaningful relevance to the use of MCL. Moreover, lines 274-278 are not justified. The authors have not provided any information about what the actual cell viability was in the experiment, and as such, simply speculating that this amount of loss probably did not occur, is not an appropriate argument. In the end, without CFU and viability information, the data are not trustworthy.

Another point that was previously raised was related to the wide-ranging conclusions about pathways based on just a very few differences of single metabolites. Again, it should be noted that there are many, many steps between these metabolites and, for example, "cytokine secretion," and there are no experiments that directly evaluate cytokine secretion. The conclusions remain to be unsupported by the data and are considered highly speculative.

Lines 97-100: The authors state that the findings help differentiate between B. canis and other Brucella species; however, no information is provided to support this claim.

As noted in the original submission, the legend of Fig. 1 is oddly organized. It would be more beneficial to the reader to have the groups ordered sequentially.

Reviewer #2: The authors´have improved results and discussion sections to relate accordingly to their results. A major concern was the lack of experimental data regarding intracellular trafficking and infection status at the timepoints of sampling for metabolic analysis. However the authors´explicitly express that this was not performed and include an indicator that is does not replace infetion status data but at least includes a control or analysis to assure that differences are not due to an internal variable between replicates and infected vrs control conditions.

Regarding figures some minor details

Figure 1. The legend is not in order, please check that the diffrent conditions and timepoints be in order

FIgure 2. I would eliminate numerical data on the heat map, it makes it difficult to compare if replicates, or infected vrs control have similar or different patterns

Figure 3-7. The bar graphs is not clear what they represent and the figure legends doest not give clarity on this. Improve clarity for interpretation.

**********

6. PLOS authors have the option to publish the peer review history of their article (what does this mean?). If published, this will include your full peer review and any attached files.

If you choose “no”, your identity will remain anonymous but your review may still be made public.

**Do you want your identity to be public for this peer review?**  For information about this choice, including consent withdrawal, please see our Privacy Policy.

Reviewer #1: No

Reviewer #2: No

**********

[NOTE: If reviewer comments were submitted as an attachment file, they will be attached to this email and accessible via the submission site. Please log into your account, locate the manuscript record, and check for the action link "View Attachments". If this link does not appear, there are no attachment files.]

To ensure your figures meet our technical requirements, please review our figure guidelines: https://journals.plos.org/plosone/s/figures

You may also use PLOS’s free figure tool, NAAS, to help you prepare publication quality figures: https://journals.plos.org/plosone/s/figures#loc-tools-for-figure-preparation.

NAAS will assess whether your figures meet our technical requirements by comparing each figure against our figure specifications.

# Author comment: Author response to Decision Letter 1

**DOI:** 10.1371/journal.pone.0353470.r003

15 May 2026

Response to Reviewers

Manuscript title: Targeted metabolomic profiling of RAW 264.7 cells infected with Brucella canis identifies time-dependent metabolic changes

Manuscript ID: PONE-D-26-09735

Dear Editor,

We sincerely thank the Editor and Reviewers for their careful evaluation and constructive comments. We greatly appreciate the thoughtful suggestions, which substantially improved the clarity, rigor, and interpretation of the manuscript. In response, we extensively revised the manuscript to address the concerns raised. All changes are highlighted in red in the revised manuscript.

Reviewer #1

Comment:

The authors have not provided information regarding macrophage viability or infection efficiency. The MCL calculation is not convincing. Without CFU and viability information, the data are not trustworthy.

Response:

We thank the reviewer for this important comment. In the revised manuscript, we further moderated the interpretation of the Minimum Cell Loss (MCL) analysis and clarified that it is presented only as exploratory contextual information rather than as a substitute for direct infection-efficiency or viability measurements.

In addition, the limitation paragraph and conclusion section were revised to further emphasize the descriptive and hypothesis-generating nature of the present metabolomics analysis.

These revisions were implemented in the Results section (Page 11, Lines 235-240), Limitation section (Page 16, Lines 341-351), and Conclusion section (Page 16, Lines 354-362), highlighted in the revised manuscript.

Comment:

The conclusions regarding pathways and cytokine secretion remain speculative.

Response:

We agree with the reviewer that some interpretations in the original manuscript were overly speculative. In the revised manuscript, statements implying direct effects on cytokine secretion, extracellular-matrix remodeling, neurotransmission, oxidative stress, or pathway activation were revised or removed unless directly supported by the experimental data.

The manuscript now consistently emphasizes that the findings represent descriptive metabolite-level associations and hypothesis-generating observations rather than definitive mechanistic conclusions.

These revisions were implemented in the Results section (Page 10, Lines 217-219) and Discussion section (Page 13, Lines 284-295 and Page 15, Lines 320-328), highlighted in the revised manuscript.

Comment:

Lines 97–100: The authors state that the findings help differentiate between B. canis and other Brucella species; however, no information is provided to support this claim.

Response:

We thank the reviewer for this helpful comment. The Introduction section was revised to remove unsupported comparative claims regarding differentiation from other Brucella species. The revised text now more conservatively states that the present study may provide a preliminary framework for future comparative investigations.

This revision was implemented in the Introduction section (Page 5, Lines 97–100), highlighted in the revised manuscript.

Comment:

The legend of Fig. 1 is oddly organized.

Response:

We thank the reviewer for this helpful suggestion. The Figure 1 legend was reorganized to improve the sequential ordering of experimental conditions and time points and enhance readability.

This revision was implemented in Figure 1 and its legend, highlighted in the revised manuscript.

Reviewer #2

Comment:

A major concern was the lack of experimental data regarding intracellular trafficking and infection status at the timepoints of sampling.

Response:

We appreciate this important comment. As suggested, the revised manuscript now explicitly acknowledges that intracellular bacterial burden, infection efficiency, and macrophage viability were not directly quantified during the experiment. We also clarified that the MCL analysis was included only as exploratory contextual information and does not replace direct infection-status measurements.

These revisions were implemented in the Results section (Page 11, Lines 235-240), Limitation paragraph (Page 16, Lines 341-351), and Conclusion section (Page 16, Lines 354-362), highlighted in the revised manuscript.

Comment:

Figure 2. I would eliminate numerical data on the heat map.

Response:

We thank the reviewer for this valuable suggestion. To improve readability and facilitate visual comparison across conditions and replicates, numerical annotations were removed from the heatmap in Figure 2 while preserving the original clustering structure and color scaling.

This revision was implemented in Figure 2 and its legend (Page 25, Lines 504-505), highlighted in the revised manuscript.

Comment:

Figure 3–7. The bar graphs are not clear what they represent and the figure legends do not give clarity on this.

Response:

We appreciate this helpful suggestion. The legends for Figures 3–7 were revised to clarify what the plotted bars represent, including explicit descriptions of mean metabolite abundance, biological replicates, and standard deviation error bars.

These revisions were implemented in the Figure Legends section (Pages 25–26, Lines 511-538), highlighted in the revised manuscript.

We believe that these revisions have substantially improved the clarity, transparency, and scientific rigor of the manuscript. We sincerely thank the reviewers for their constructive comments and hope that the revised manuscript is now suitable for reconsideration.

Attachment Submitted filename: Response to Reviewers 0512 (1).pdf

# Peer review: Decision Letter 1

**DOI:** 10.1371/journal.pone.0353470.r004

**Authors:** Roy Martin Roop II

15 Jun 2026

PONE-D-26-09735R1

Targeted metabolomic profiling of RAW 264.7 cells infected with  *Brucella canis*   identifies time-dependent metabolic changes

PLOS One

Dear Dr. Yoo,

Thank you for submitting your manuscript to PLOS ONE. After careful consideration, we feel that it has merit but does not fully meet PLOS ONE’s publication criteria as it currently stands. Therefore, we invite you to submit a revised version of the manuscript that addresses the points raised during the review process.

First of all, let me sincerely apologize for the delay in getting your revised manuscript reviewed and an editorial decision made! Nevertheless, as you can see from their comments below Reviewer 2 still has significant concerns that would need to be addressed before the paper will be judged as being suitable for publication in PLOS ONE. If  you would like to submit a second revision that focuses on addressing these specific issues I will be happy to review it.

If you choose to take this route, please submit your revised manuscript by Jul 30 2026 11:59PM. If you will need more time than this to complete your revisions, please reply to this message or contact the journal office at plosone@plos.org. When you're ready to submit your revision, log on to https://www.editorialmanager.com/pone/ and select the 'Submissions Needing Revision' folder to locate your manuscript file.

Please include the following items when submitting your revised manuscript:

- A letter that responds to each point raised by the academic editor and reviewer(s). You should upload this letter as a separate file labeled 'Response to Reviewers'.
- A marked-up copy of your manuscript that highlights changes made to the original version. You should upload this as a separate file labeled 'Revised Manuscript with Track Changes'.
- An unmarked version of your revised paper without tracked changes. You should upload this as a separate file labeled 'Manuscript'.

If you would like to make changes to your financial disclosure, please include your updated statement in your cover letter. Guidelines for resubmitting your figure files are available below the reviewer comments at the end of this letter.

If applicable, we recommend that you deposit your laboratory protocols in protocols.io to enhance the reproducibility of your results. Protocols.io assigns your protocol its own identifier (DOI) so that it can be cited independently in the future. For instructions see: https://journals.plos.org/plosone/s/submission-guidelines#loc-laboratory-protocols. Additionally, PLOS ONE offers an option for publishing peer-reviewed Lab Protocol articles, which describe protocols hosted on protocols.io. Read more information on sharing protocols at https://plos.org/protocols?utm_medium=editorial-email&utm_source=authorletters&utm_campaign=protocols.

As the corresponding author, your ORCID iD is verified in the submission system and will appear in the published article. PLOS supports the use of ORCID, and we encourage all coauthors to register for an ORCID iD and use it as well. Please encourage your coauthors to verify their ORCID iD within the submission system before final acceptance, as unverified ORCID iDs will not appear in the published article. *Only*  the individual author can complete the verification step; PLOS staff *cannot*  verify ORCID iDs on behalf of authors.

Again, please accept my apologies for the delay in getting your paper evaluated!

Sincerely,

R. Martin Roop II, Ph.D.

Academic Editor

PLOS One

Journal Requirements:

If the reviewer comments include a recommendation to cite specific previously published works, please review and evaluate these publications to determine whether they are relevant and should be cited. There is no requirement to cite these works unless the editor has indicated otherwise.

[Note: HTML markup is below. Please do not edit.]

Reviewers' comments:

Reviewer's Responses to Questions

**Comments to the Author**

1. If the authors have adequately addressed your comments raised in a previous round of review and you feel that this manuscript is now acceptable for publication, you may indicate that here to bypass the “Comments to the Author” section, enter your conflict of interest statement in the “Confidential to Editor” section, and submit your "Accept" recommendation.

Reviewer #1: All comments have been addressed

Reviewer #2: (No Response)

**********

2. Is the manuscript technically sound, and do the data support the conclusions?

The manuscript must describe a technically sound piece of scientific research with data that supports the conclusions. Experiments must have been conducted rigorously, with appropriate controls, replication, and sample sizes. The conclusions must be drawn appropriately based on the data presented.

Reviewer #1: Yes

Reviewer #2: Yes

**********

3. Has the statistical analysis been performed appropriately and rigorously?

Reviewer #1: Yes

Reviewer #2: Yes

**********

4. Have the authors made all data underlying the findings in their manuscript fully available?

The PLOS Data policy requires authors to make all data underlying the findings described in their manuscript fully available without restriction, with rare exception (please refer to the Data Availability Statement in the manuscript PDF file). The data should be provided as part of the manuscript or its supporting information, or deposited to a public repository. For example, in addition to summary statistics, the data points behind means, medians and variance measures should be available. If there are restrictions on publicly sharing data—e.g. participant privacy or use of data from a third party—those must be specified.

Reviewer #1: Yes

Reviewer #2: Yes

**********

5. Is the manuscript presented in an intelligible fashion and written in standard English?

PLOS ONE does not copyedit accepted manuscripts, so the language in submitted articles must be clear, correct, and unambiguous. Any typographical or grammatical errors should be corrected at revision, so please note any specific errors here.

Reviewer #1: Yes

Reviewer #2: Yes

**********

6. Review Comments to the Author

Please use the space provided to explain your answers to the questions above. You may also include additional comments for the author, including concerns about dual publication, research ethics, or publication ethics. (Please upload your review as an attachment if it exceeds 20,000 characters)

Reviewer #1: (No Response)

Reviewer #2: The authors have substantially improved the manuscript, in terms of clarity, experimental design description and conclusions according to the results. This study is relevant and contributes to the global understanding of the host cell response to B. canis interaction and provide valuable information for an understudied Brucella model. In my consideration the authors have to improve the following observations for clarity:

-The authors include a justification and reasoning for the lack of experimental data regarding infection kinetics for each metabolic timepoint. However the authors have to emphasize and clearly state that the infection outcome is not known and response can go from just interaction, to infection or both. Either way there is a metabolic response as clearly seen in the results, so a biological explanation and not just a justification is required to overcome this limitation. As reported in various studies and personal experience, for B. canis succesful infections (repliactive phenotype) depend of a narrow physiological state of the bacteria and sometimes difficult to achieve, it is common to obtain the non replicative phenotype were bacteria are just attached to the cell. I recommended including to the limitation of the missing infection data a biological explanation that highlights why interpretation has to be limited to a broader potential interaction and not to specific cell response events exclusively from B. canis succesful infection.

- Regarding figures 3 to 7. The authors have included information that improves clarity for the interpretation of the figures and the text. However it is still not clear enough. What do the color of the bars represent? each control and treatment? Do the color maintain the same interpretation than in figure 1? If this is the correct interpretation, the authors should improve the order of the bars for a correct undesrtanding, according to the different timepoints for treatments and controls. If this is not the correct interpreation precise description in the results section, figures and figures legends to avoid erroneous interpreattion of the results.

**********

7. PLOS authors have the option to publish the peer review history of their article (what does this mean?). If published, this will include your full peer review and any attached files.

If you choose “no”, your identity will remain anonymous but your review may still be made public.

**Do you want your identity to be public for this peer review?**  For information about this choice, including consent withdrawal, please see our Privacy Policy.

Reviewer #1: No

Reviewer #2: No

**********

[NOTE: If reviewer comments were submitted as an attachment file, they will be attached to this email and accessible via the submission site. Please log into your account, locate the manuscript record, and check for the action link "View Attachments". If this link does not appear, there are no attachment files.]

To ensure your figures meet our technical requirements, please review our figure guidelines: https://journals.plos.org/plosone/s/figures

You may also use PLOS’s free figure tool, NAAS, to help you prepare publication quality figures: https://journals.plos.org/plosone/s/figures#loc-tools-for-figure-preparation.

NAAS will assess whether your figures meet our technical requirements by comparing each figure against our figure specifications.

# Author comment: Author response to Decision Letter 2

**DOI:** 10.1371/journal.pone.0353470.r005

21 Jun 2026

Response to Reviewers

Manuscript title: Targeted metabolomic profiling of RAW 264.7 cells infected with Brucella canis identifies time-dependent metabolic changes

Manuscript ID: PONE-D-26-09735R1

Dear Editor,

We sincerely thank the Editor and Reviewers for their careful evaluation and constructive comments. We greatly appreciate the thoughtful suggestions, which substantially improved the clarity, rigor, and interpretation of the manuscript. In response, we extensively revised the manuscript to address the concerns raised. All changes are highlighted in red in the revised manuscript.

Reviewer #2

Comment:

The authors should more clearly emphasize that infection outcome was not determined at each metabolic sampling time point and that the observed responses may reflect bacterial attachment, host–pathogen interaction, intracellular infection, or a combination of these processes.

Response:

We thank the reviewer for this insightful comment. We agree that, because infection status was not directly characterized at each sampling time point, the observed metabolic responses cannot be attributed specifically to successful intracellular replication of B. canis. In response, we expanded the limitation section to provide additional biological context regarding the interpretation of the observed metabolite profiles. Specifically, we clarified that Brucella–macrophage interactions may encompass multiple stages, including bacterial attachment, intracellular uptake, persistence, and replication, and that productive intracellular infection depends on both bacterial and host physiological conditions [7, 20]. Therefore, the measured metabolite profiles may reflect a combination of bacterial attachment, host–pathogen interaction, and intracellular infection processes, and the relative contribution of these interaction states could not be distinguished in the present study. We further clarified that the findings should be interpreted as metabolic responses associated with B. canis exposure rather than as specific signatures of intracellular replication.

This revision was implemented in the Limitation section (Page 15-16, Lines 338–347), highlighted in the revised manuscript.

Comment:

Regarding Figures 3–7, the meaning of the inset bar plots and their color coding is not sufficiently clear.

Response:

We appreciate this helpful suggestion. The legends of Figures 3–7 were revised to clarify the interpretation of the inset bar plots. Specifically, we added descriptions indicating that the inset bar plots represent mean metabolite abundance across experimental groups and time points and clarified the chronological order of the displayed groups (0h-Control, 2h-Control, 12h-Control, 24h-Control, 2h-Treatment, 12h-Treatment, and 24h-Treatment).

These revisions were implemented in the Figure Legends section (Pages 25–27), highlighted in the revised manuscript.

We believe that these revisions have substantially improved the clarity, transparency, and scientific rigor of the manuscript. We sincerely thank the reviewers for their constructive comments and hope that the revised manuscript is now suitable for reconsideration.

Attachment Submitted filename: Responses to Reviewers -PONE-D-26-09735R1.pdf

# Editor report: Decision Letter 2

**DOI:** 10.1371/journal.pone.0353470.r006

**Authors:** Roy Martin Roop II

24 Jun 2026

Targeted metabolomic profiling of RAW 264.7 cells infected with  *Brucella canis*   identifies time-dependent metabolic changes

PONE-D-26-09735R2

Dear Dr. Yoo,

Thank you for your rapid response to the previous review, and again I apologize for the delay in getting your paper processed! I'm glad to inform you that your manuscript is now scientifically suitable for publication and will be formally accepted for publication once it meets all outstanding technical requirements.

Within one week, you’ll receive an e-mail detailing the required amendments. When these have been addressed, you’ll receive a formal acceptance letter and your manuscript will be scheduled for publication.

An invoice will be generated when your article is formally accepted. Please note, if your institution has a publishing partnership with PLOS and your article meets the relevant criteria, all or part of your publication costs will be covered. Please make sure your user information is up-to-date by logging into Editorial Manager at Editorial Manager® and clicking the ‘Update My Information' link at the top of the page. For questions related to billing, please contact billing support.

If your institution or institutions have a press office, please notify them about your upcoming paper to help maximize its impact. If they’ll be preparing press materials, please inform our press team as soon as possible -- no later than 48 hours after receiving the formal acceptance. Your manuscript will remain under strict press embargo until 2 pm Eastern Time on the date of publication. For more information, please contact onepress@plos.org.

Sincerely,

R. Martin Roop II, Ph.D.

Academic Editor

*PLOS One*

Additional Editor Comments (optional):

Reviewers' comments:

# Editor report: Acceptance letter

**DOI:** 10.1371/journal.pone.0353470.r007

**Authors:** Roy Martin Roop II

PONE-D-26-09735R2

PLOS One

Dear Dr. Yoo,

I'm pleased to inform you that your manuscript has been deemed suitable for publication in PLOS One. Congratulations! Your manuscript is now being handed over to our production team.

At this stage, our production department will prepare your paper for publication. This includes ensuring the following:

* All references, tables, and figures are properly cited

* All relevant supporting information is included in the manuscript submission,

* There are no issues that prevent the paper from being properly typeset

You will receive further instructions from the production team, including instructions on how to review your proof when it is ready. Please keep in mind that we are working through a large volume of accepted articles, so please give us a few days to review your paper and let you know the next and final steps.

Lastly, if your institution or institutions have a press office, please let them know about your upcoming paper now to help maximize its impact. If they'll be preparing press materials, please inform our press team within the next 48 hours. Your manuscript will remain under strict press embargo until 2 pm Eastern Time on the date of publication. For more information, please contact onepress@plos.org.

You will receive an invoice from PLOS for your publication fee after your manuscript has reached the completed accept phase. If you receive an email requesting payment before acceptance or for any other service, this may be a phishing scheme. Learn how to identify phishing emails and protect your accounts at https://explore.plos.org/phishing.

If we can help with anything else, please email us at customercare@plos.org.

Thank you for submitting your work to PLOS ONE and supporting open access.

Kind regards,

PLOS ONE Editorial Office Staff

on behalf of

Dr. Roy Martin Roop II

Academic Editor

PLOS One
